# The Regulation of Reverse Cholesterol Transport and Cellular Cholesterol Homeostasis by MicroRNAs

**DOI:** 10.3390/biology4030494

**Published:** 2015-07-28

**Authors:** Diana M. DiMarco, Maria Luz Fernandez

**Affiliations:** Department of Nutritional Sciences, University of Connecticut, Storrs, CT 06269, USA; E-Mail: diana.dimarco@uconn.edu

**Keywords:** miRNA, plasma lipoproteins, reverse cholesterol transport, cholesterol homeostasis

## Abstract

MicroRNAs (miRNAs) are small, non-coding RNAs that have the ability to post-transcriptionally regulate gene expression. Hundreds of miRNAs have been identified in humans and they are involved in the regulation of almost every process, including cholesterol transport, metabolism, and maintenance of cholesterol homeostasis. Because of their small size and their ability to very specifically regulate gene expression, miRNAs are attractive targets for the regulation of dyslipidemias and other lipid-related disorders. However, the complex interactions between miRNAs, transcription factors, and gene expression raise great potential for side effects as a result of miRNA overexpression or inhibition. Many dietary components can also target specific miRNAs, altering the expression of downstream genes. Therefore, much more research is necessary to fully understand the role(s) of each miRNA in the body and how they may be impacted by diet and health. The present review aims to summarize the known roles of miRNAs in the regulation of reverse cholesterol transport and the maintenance of cholesterol homeostasis, as well as the potential clinical consequences of their manipulation.

## 1. Introduction

MicroRNAs (miRNAs) are small non-coding RNAs (~22 nucleotides) that are typically encoded within the introns of other genes [1]. MiRNAs are synthesized as immature pro-miRNAs that are cleaved into pre-miRNA by Drosha ribonuclease type III. The pre-miRNA transcript is then further cleaved into its active form by Dicer 1 ribonuclease type III [2,3]. Mature miRNAs interact with Argonaute proteins to form an RNA silencing complex that interacts with the 3'-untranslated region (3' UTR) of target mRNA transcripts via approximate base pair matching to post-transcriptionally regulate gene expression [3,4]. Such miRNA–miRNA interactions either repress translation or promote cleavage of the mRNA transcript [1]. MiRNAs are unavoidably transcribed as a duplex, though often one strand is much less stable and therefore does not accumulate [3]. This less stable strand is called the passenger strand and is indicated by a-3p. Most passenger strands have minimal ability to interact with mRNA, though some are now being recognized as having regulatory capacities [5,6].

Recent research has identified a role of miRNAs in numerous processes, including growth and development [7], oncogenesis [8], and lipid metabolism [9]. Because of their small size and ability to interact with mRNA transcripts without exact base-pair matching, miRNAs are highly pleiotropic; they can target multiple genes while, conversely, many mRNAs are regulated by numerous miRNAs [10]. Therefore, the role of miRNAs is highly complex and only now beginning to be fully elucidated.

Elevated concentrations of plasma LDL-cholesterol (LDL-C), as well as dyslipidemias associated with low HDL-cholesterol (HDL-C) and high plasma triglycerides (TG), are well-established risk factors for cardiovascular disease (CVD) [11]. CVD is the leading cause of death in the United States [12], and therefore any interventions aimed at reducing these dyslipidemias are of great interest. Free cholesterol (FC) and TG are secreted by the liver in the form of VLDL with the help of the chaperone protein microsomal triglyceride transfer protein (MTP) [13]. In the bloodstream, TG are hydrolyzed from VLDL by the action of lipoprotein lipase (LPL) and delivered to tissues [14]. As this occurs, VLDL is delipidated and becomes LDL [14,15]. LDL is the major deliverer of cholesterol to tissues, though it is also highly prone to modification, which predisposes it to unregulated uptake into macrophages leading to the formation of atherosclerotic lesions [16].

Conversely, reverse cholesterol transport (RCT) is the process by which excess cholesterol is effluxed from cells into HDL particles and returned to the liver for excretion from the body [17,18]. This process is crucial for the prevention of lipid accumulation, particularly in atherosclerotic lesions. RCT is a complex process that relies upon the association of apolipoprotein A1 (ApoA1) with ATP-binding cassette transporter A1 (ABCA1), which effluxes cholesterol to ApoA1 to form a nascent HDL particle. ATP-binding cassette transporter G1 (ABCG1) further effluxes cholesterol to nascent HDL, eventually forming a mature HDL particle [17,18]. HDL then returns to the liver and is taken up by scavenger receptor B1 (SRB1), where the cholesterol is targeted for elimination via catabolism to bile acids or direct biliary secretion [17]. Therefore, RCT is considered an atheroprotective process.

**Figure 1:**
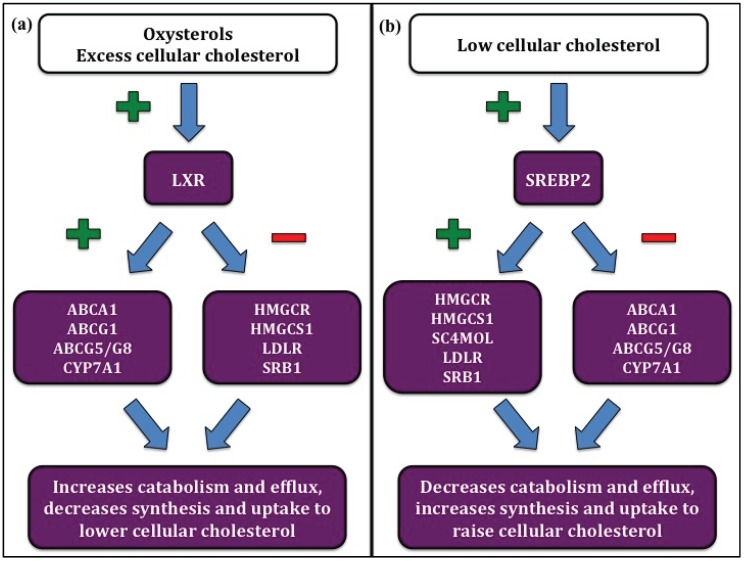
Regulation of cellular cholesterol homeostasis. (**a**) Liver X receptor (LXR) is activated by excess cellular cholesterol. LXR activation increases cholesterol efflux and catabolism by upregulating ATP binding cassette A1 (ABCA1), ATP binding cassette G1 (ABCG1), ATP binding cassette G5/G8 (ABCG5/G8), and cholesterol 7α hydroxylase (CYP7A1), and decreases cholesterol synthesis and uptake by suppressing 3-hydroxy-3-methylglutaryl CoA reductase (HMGCoA-R), 3-hydroxy-3-methylglutaryl CoA synthase 1 (HMGCoA-S1), LDL receptor (LDLR), and scavenger receptor B1 (SRB1). (**b**) Sterol regulatory element-binding protein 2 (SREBP2) is upregulated by low cellular cholesterol. SREBP2 upregulates cholesterol synthesis and uptake by increasing HMGCoA-R, HMGCoA-S1, methylsterol monoxygenase (SC4MOL), LDLR, and SRB1, and decreases cholesterol efflux and catabolism by suppressing ABCA1, ABCG1, ABCG5/G8, and CYP7A1.

The rates of forward and reverse cholesterol transport and cholesterol synthesis are under tight control in order to maintain cellular cholesterol balance. Sterol regulatory element-binding protein 2 (SREBP2) is a transcription factor that is activated by low cellular cholesterol and acts to increase cellular cholesterol levels by facilitating synthesis and uptake and decreasing efflux (Figure 1a) [19,20]. Conversely, the liver X receptor (LXR) family of transcription factors is activated in cases of cholesterol excess and decreases cellular cholesterol by upregulating efflux and decreasing synthesis and uptake (Figure 1b) [21,22].

Though the process of cholesterol transport is well characterized, the precise regulation of each of the many genes involved is still not fully understood. Furthermore, existing therapeutic strategies are known to result in numerous side effects [23,24]. MiRNAs exhibit the potential for precise regulation of lipid metabolism with potentially minimal side effects. The present review therefore summarizes the many miRNAs implicated in regulation of cholesterol homeostasis, metabolism, and transport, and the potential clinical consequences of their manipulation.

## 2. Regulation of Plasma Lipoproteins by miRNAs

MicroRNA (miR)-122 is the most prevalent miRNA in the liver and regulates expression of various lipogenic genes [25]. Inhibition of this miRNA in mice led to reduced expression of these genes and subsequent decreases in lipogenesis and, therefore, plasma cholesterol [26]. There was also a reduction in hepatic lipid accumulation with miR-122 inhibition. The authors hypothesize this may be due to an miR-122-mediated increase in fatty acid oxidation [26], suggesting this as a strategy to improve overall lipid metabolism. Nevertheless, more *in vivo* trials should be conducted. As mice transport most of their cholesterol in HDL (rather than LDL, as humans do), it is also important that subsequent *in vivo* trials be conducted in animals with lipoprotein profiles more similar to that of humans in order to fully understand the clinical potential of anti-miR-22 therapy.

Formation of VLDL in the liver requires the presence of MTP, which facilitates the association of lipids with apolipoprotein B (ApoB) [13]. Recently, MTP was identified as a target of miR-30c [27]. Indeed, miR-30c decreased MTP mRNA, activity, and ApoB secretion (but not synthesis) *in vitro* [27]. *In vivo*, miR-30c expression reduced hepatic MTP expression and reduced plasma concentrations of triglyceride-rich lipoproteins (TRL). Unlike synthetic MTP inhibitors, miR-30c did not cause hepatic steatosis, likely due to the simultaneous downregulation of genes involved in *de novo* lipid synthesis, such as lysophosphatidylglycerol acyltransferase 1 (LPGAT1) [27]. MiR-30c treatment also resulted in fewer atherosclerotic lesions and smaller lesion area compared with anti-miR-30c-treated animals [27]. Therefore, miR-30c may hold promise as a VLDL, LDL, and/or TG lowering agent, though further trials and a more comprehensive understanding of the systemic impacts of miR-30c are necessary.

Conversely, miR-27b may regulate plasma HDL cholesterol. In a study published by Vickers *et al*. [28], miR-27b suppressed miRNA and protein expression of Angiopoietin-like 3 (ANGPTL3) *in vitro*. ANGPTL3 is responsible for regulation of endothelial lipase (EL), an enzyme which is thought to promote HDL catabolism [29]. Because EL reduces plasma HDL cholesterol, the hypothesis is that miR-27b overexpression should suppress ANGPTL3 and, therefore, EL. However, in a model of elevated miR-27b, ANGPTL was only slightly reduced [28]. Perhaps physiological levels of miR-27b are not high enough to facilitate significant suppression or perhaps there are other components of this pathway that have not yet been identified. Clearly, more research is needed to determine if and how miR-27b impacts plasma HDL.

Not only are lipoprotein formation and concentration regulated by miRNAs, but emerging data shows that lipoproteins are vehicles for miRNAs [30,31]. HDL is the primary lipoprotein involved in miRNA transport, with miR-223 being the most abundant miRNA [31]. Many other miRNAs are found on both HDL and LDL, albeit in much smaller amounts [30,31]. Interestingly, the lipoprotein miRNA profile appears to differ between healthy and diseased individuals, with an increase in pro-atherogenic miRNAs seen on HDL isolated from individuals with familial hypercholesterolemia [30].

Uptake assays show mixed results, with some studies showing minimal uptake of miRNAs from HDL [31] while others suggest that uptake via SRB1 leads to alterations of gene expression in the recipient cells [30]. That miR-223 is the main miRNA found on HDL is particularly interesting because, as discussed in more detail below, miR-223 is also capable of regulating SRB1 expression [32,33]. Therefore, miR-223 may be able to regulate its own cellular uptake. It is also possible that miRNAs may account for some of the pro- or anti-atherogenic characteristics of lipoproteins and may be something to take into account when assessing CVD risk.

## 3. Regulation of Cellular Cholesterol Homeostasis by miRNAs

As discussed briefly above, low intracellular cholesterol levels trigger SREBP2 activation and subsequent regulation of cholesterol synthesis, uptake, and efflux to restore homeostasis (Figure 1b) [19,20]. Similar to other transcription factors, SREBP2 has the ability to autoregulate, and it was recently shown that this ability might be mediated by miRNAs. In fact, *in vitro* treatment with SREBP2 directly increased transcription of an miRNA locus encoding miR-96, miR-182, and miR-183, all three of which go on to indirectly increase SREBP2 expression [34].

SREBP2 is also regulated by other transcription factors in a process involving miR-185 [35]. Indeed, miR-185 decreased SREBP2 mRNA and protein expression *in vitro*, leading to a downstream decrease in SREBP2-responsive genes, including LDL receptor (LDLR) [35], resulting in a subsequent decrease in cellular LDL uptake. To draw further insight into this pathway, the authors showed that miR-185 expression is negatively regulated by another member of the SREBP family, SREBP1c [35]. Furthermore, SREBP1c is activated by LXR [21]. Therefore, elevated cellular cholesterol activates LXR, which upregulates SREBP1c, which in turn increases expression of miR-185 to suppress SREBP2 and its downstream targets, leading to a reduction in LDLR-mediated cholesterol uptake [35].

MiR-613 expression is also upregulated *in vitro* by SREBP1c upon its activation by LXR, which then leads to the suppression of LXR in a negative, autoregulatory feedback loop [36]. LXR suppresses expression of genes involved in synthesis and uptake, including the inducible degrader of LDLR (IDOL), which decreases uptake of cholesterol via LDLR [37]. Because miR-613 represses LXR, it appears to be involved in the resolution of LXR-mediated changes to the cell upon restoration of homeostasis.

MiR-1 and miR-206 were also recently shown to suppress LXRα *in vitro* [38]. This particular study examined only downstream genes involved in regulation of lipogenesis, but as LXR is a known regulator of cholesterol-related genes, miR-1 and miR-206 may also be involved in regulation of cholesterol homeostasis through their ability to suppress LXRα and, subsequently, downstream target genes involved in cholesterol synthesis, transport, and uptake (Figure 1a).

## 4. Regulation of Cholesterol Synthesis by miRNAs

Though most miRNAs that have thus far been discussed regulate cholesterol influx and efflux, a growing number also regulate cholesterol biosynthesis. One such miRNA is miR-223 [33]. MiR-223 overexpression *in vitro* repressed mRNA expression of 3-hydroxy-3-methylglutaryl-coA-synthase 1 (HMGCoA-S1) and methylsterol monoxygenase 1 (SC4MOL), two genes in the cholesterol biosynthetic pathway [33]. Even under hypocholesterolemic conditions, treatment with miR-223 suppressed HMGCoA-S1 and SC4MOL expression, suggesting that perhaps its expression is sensitive to cellular cholesterol concentrations. However, expression of 3-hydroxy-3-methylglutaryl-coA reductase (HMGCoA-R) was increased with miR-223 overexpression, indicating a more complicated pathway of miR-223-mediated regulation of cholesterol biosynthesis [33].

Recently, miR-27b and miR-185 were also shown to regulate expression of HMGCoA-R [28,35], although suppression of HMGCoA-R by miR-27b *in vitro* did not reach statistical significance (*p* = 0.06) [28]. In a second study, HMGCoA-R upregulation by cholesterol depletion was attenuated by miR-185 overexpression [35]. Overexpression of miR-185 under normocholesterolemic conditions did not significantly alter HMGCoA-R expression levels [35], though, which suggests that miR-185 may be involved in the fine-tuning of HMGCoA-R expression in hypocholesterolemic conditions.

The signal transducer and activator of transcription (STAT) family of transcription factors are most well known for their roles in cancer progression and immune signaling [39,40]. Recent studies have uncovered a possible link between abnormal cholesterol levels and the risk for some cancers, with STAT6 being involved in this process [41]. For example, STAT6 binds the promoter region of certain miRNAs *in vitro*, including miR-197 [42]. Knockdown of miR-197 revealed an increase in forkhead box J2 (FOXJ2), a transcription factor that regulates cholesterol synthetic genes. Further studies showed that treatment of lung cancer cells with miR-197 led to decreases in expression of HMGCoA-R, HMGCoA-S1, and isopentenyl-diphosphate delta isomerase 1 (IDI1) [42]. All of these genes possess FOXJ2 binding sites. Indeed, STAT6 silencing decreases miR-197 expression, leading to increased FOXJ2 expression and subsequent increases in HMGCoA-R, HMGCoA-S1, IDI1, and cholesterol synthesis [42].

Though this particular study was aimed at further understanding cancer biology, the results illustrate the complex network of genes, transcription factors, and miRNAs that work together to mediate gene expression in many potentially overlapping pathways.

## 5. Regulation of Macrophage Cholesterol Uptake by miRNAs

RCT is the process of removing excess cholesterol from cells, particularly macrophages, and returning it to the liver for excretion. An obvious prerequisite to this process is uptake of cholesterol by macrophages. This process is facilitated primarily via CD36, SRB1, and LPL [43,44,45]. LPL is expressed on the surface of endothelial cells and facilitates uptake of lipids from lipoproteins into the cell [14]. This process is typically considered anti-atherogenic; however, retention of lipoproteins by LPL increases the likelihood of modification and uptake by macrophages [45].

MiR-27 was discussed above with regard to its potential ability to regulate HDL catabolism and cholesterol synthesis. However, miR-27 also regulates cholesterol homeostasis by suppressing macrophage cholesterol uptake [46]. Indeed, *in vitro* treatment with miR-27 suppressed LPL mRNA and protein expression while inhibition of miR-27 yielded the opposite effect. MiR-27 also suppressed CD36 expression, though interestingly, CD36 does not contain an miR-27 binding site [46] suggesting that this specific regulation might be indirect. Regardless, miR-27-mediated suppression of LPL and CD36 may reduce macrophage cholesterol uptake and attenuate lesion formation. 

Lastly, miR-96, miR-185, and miR-223 have all been shown to suppress SRB1 expression *in vitro* and, therefore, regulate macrophage cholesterol uptake [32,33].

## 6. Regulation of Cholesterol Esterification by miRNAs

Esterification of cholesterol is necessary for maintenance of cholesterol in the cell, as well as to prevent toxicities associated with accumulation of FC. Esterification of FC is catalyzed by the acetyl coA:cholesterol acyl transferase (ACAT) family of enzymes. Cholesterol that has not been esterified can be incorporated into cell membranes or effluxed from the cell, but esterified cholesterol is retained. This is particularly important for retention of cholesterol in HDL, but a more concerning implication is the role of ACAT1 in macrophages. Increased ACAT1 activity favors retention of cholesterol ester (CE) in the macrophage, promoting foam cell development [47].

MiR-9 is a recently identified regulator of ACAT1 expression [47]. Indeed, transfection of macrophages with miR-9 suppressed ACAT1 protein, but not mRNA, expression. Macrophage TC, CE, and lipid droplet size were also reduced with ACAT1 suppression [47]. This suggests the possibility of miR-9-mediated ACAT1 suppression as a therapeutic strategy to reduce foam cell formation. In addition to its numerous other target genes, miR-27 also decreased ACAT1 mRNA and protein expression *in vitro* [46].

## 7. Regulation of Cholesterol Efflux by miRNAs

The most common targets of miRNAs in the process of RCT appear to be the cholesterol efflux transporters. Recent studies have uncovered numerous miRNAs that regulate ABCA1 expression and subsequent efflux of cholesterol to ApoA1. ABCA1 has an unusually long 3' UTR that harbors many miRNA binding sites, subjecting it to regulation by many miRNAs [48]. These miRNAs, and others, are discussed in detail below.

### 7.1. Regulation of ABCA1 by miRNAs

*In vitro*, numerous miRNAs regulate ABCA1 expression and, therefore, cholesterol efflux to ApoA1. Treatment with miR-27a/b [46], miR-128 [49], miR-145 [50], miR-302 [51], and miR-758 [48] directly suppressed ABCA1 by binding to its 3' UTR and attenuated cholesterol efflux to ApoA1. Conversely, miR-223 treatment increased ABCA1 expression and cholesterol efflux to ApoA1, though by an indirect mechanism [33]. Previous research shows that ABCA1 is under the influence of two transcription factors: Sp1 and Sp3 [52]. Sp3 is an Sp1 antagonist [52]; indeed, *in vitro*, miR-223 antagonized Sp3, derepressing Sp1 and allowing for induction of ABCA1 [33]. In contrast, miR-23 inhibition *in vivo* did not alter liver ABCA1 expression despite the results achieved *in vitro* [33]. This may be attributable to the numerous other miRNAs that regulate ABCA1 expression.

*In vitro* work suggests that miR-33b-3p regulates ABCA1 indirectly, via Sp1 [6], while miR-33a-3p also regulates ABCA1 indirectly via the transcription factor steroid receptor coactivator 1 (SRC1). *In vivo*, ABCA1 suppression in macrophages by miR-19b [53] and miR-144-3p [5], and in liver by miR-144-5p [54], reduced HDL cholesterol and RCT. ABCA1 suppression manifested as increased hepatic lipid deposition [5], increased production of proinflammatory cytokines [5], and markedly increased lesion area and lesion macrophage content versus control animals [5,53]. Inhibition of miR-33 increased hepatic ABCA1 expression, plasma ApoA1 and HDL, and RCT, and decreased aortic plaque size, lesion macrophage and lipid content, and macrophage cholesterol efflux [55,56,57]. Inhibition of miR-302a also increased plasma HDL, decreased plaque formation, and promoted lesion remodeling. These results highlight the potential of miR-33 or miR-302a suppression as a strategy to promote RCT and the regression of atherosclerosis [51,55].

MiRNAs also mediate the actions of transcription factors on cholesterol-related genes. LXR activation *in vitro* decreases the expression of miR-26 [58], reversing miR-26-mediated suppression of ABCA1 and ADP-ribosylation factor-like 7 (ARL7), the transporter that carries cholesterol to the membrane to facilitate association with ABCA1 [59]. LXR activation also increases miR-144-5p, which suppresses ABCA1 and cholesterol efflux to ApoA1 [60]. Likewise, activation of peroxisome proliferator activated receptor gamma (PPARγ) decreased miR-613 expression, removing inhibition of LXR and ABCA1 and increasing macrophage cholesterol efflux [61]. Farnesoid X receptor (FXR) suppresses ABCA1 by upregulating miR-33b [62]. FXR also increases miR-144-5p expression to mediate ABCA1 suppression and cholesterol efflux [54]; perhaps miR-33b and miR-144-5p work together to mediate FXR-induced ABCA1 repression. Though they have not been studied in tandem, it also appears that LXR and FXR may be components of a feedback loop that helps maintain cholesterol balance by regulating ABCA1 expression via miR-144-5p.

With so many miRNAs in control of ABCA1 expression, it is intuitive that they may work together to regulate its expression. Indeed, multiple groups have shown that miR-33-5p and miR-144-5p have an additive effect on suppression of ABCA1 [54,60,62], while cotransfection of miR-33-5p and miR-758 further reduced ABCA1 expression *in vitro* [48]. MiR-33-5p and its passenger strand, miR-33-3p, also have an additive effect on expression of ABCA1 [6]. However, co-treatment of cells with miR-33-5p and miR-145 did not have an additive effect on ABCA1 expression [50]. It is clear that miRNA-mediated regulation of ABCA1 is very complex. In addition, some of these miRNAs act upon many genes, highlighting the difficulty of specifically targeting ABCA1 without impacting the expression of other genes. With numerous miRNAs able to bind ABCA1 at one time, there are many possible combinations, some of which may work together or in opposition to regulate ABCA1 expression. As the mechanism of regulation of each of these miRNAs is still not fully comprehended, understanding how they work together to regulate ABCA1 will take some time.

### 7.2. Regulation of ABCG1 by miRNAs

To date, much less is known about the regulation of ABCG1 by miRNAs. It is a direct target of miR-10b [63] and miR-128 [49], and is indirectly suppressed by miR-27 [46] and miR-378 [64]. Cholesterol efflux to HDL is also decreased by ABCG1 suppression [46,49,63,64]. As was seen with the regulation of ABCA1, miR-144-5p is transcribed following LXR activation and suppresses ABCG1 mRNA expression *in vitro* [54]. Interestingly, ABCG1 is a target of miR-33-5p in mice but not humans [65]. Though fewer miRNAs seem to be involved in regulation of ABCG1, increasing its expression results in increased plasma HDL. Therefore, targeting these miRNAs may be a promising option for individuals with low HDL.

## 8. Regulation of Hepatic HDL by miRNAs

Upon acquiring cholesterol from macrophages and other tissues, mature HDL returns to the liver where it is taken up by SRB1 and the cholesterol is targeted for excretion [17]. Therefore, SRB1-mediated uptake is crucial for the removal of cholesterol from the body.

MiR-223 was previously discussed for its ability to regulate numerous genes, including macrophage SRB1. MiR-223 also suppresses hepatic SRB1 expression, leading to a reduction in hepatic HDL uptake [33]. Along with miR-223, miR-96 and miR-185 were also identified as regulators of SRB1 expression [32]. Indeed, *in vitro* expression of each of the three miRNAs significantly decreased SRB1 expression and HDL uptake. In fact, expression of all three miRNAs had an additive effect on SRB1 suppression [32]. MiR-185 and miR-223 are activated by hypercholesterolemic conditions, therefore their ability to inhibit further cholesterol uptake is unsurprising. Despite its ability to suppress SRB1 expression, however, miR-96 actually increased HDL uptake, suggesting that miR-96 must target other cholesterol uptake pathways [32]. Clearly more research is needed to understand the role of miR-96 in regulation of cholesterol uptake and how these three miRNAs work together to mediate cholesterol uptake by SRB1.

## 9. Regulation of Cholesterol Catabolism by miRNAs

The final step in the process of RCT is the excretion of cholesterol from the body, a process achieved by the catabolism of cholesterol into bile acids and biliary cholesterol secretion.

Two groups recently showed that miR-33a, in addition to the previously discussed roles in regulation of cholesterol homeostasis, is also involved in regulation of bile acid metabolism [66,67]. MiR-33a overexpression *in vitro* reduced expression of cholesterol 7α hydroxylase (CYP7A1), sterol 12α hydroxylase (CYP8B1), sodium-taurocholate cotransporting polypeptide (NTCP), bile salt export pump (BSEP), ATP binding cassette transporter G5 (ABCG5), and ATP binding cassette transporter G8 (ABCG8), all of which are involved in the synthesis and secretion of bile acids in the liver [66]. Bile acid pool size was also significantly reduced. *In vivo*, mRNA expression of these proteins were reduced following miR-33a overexpression, while miR-33a inhibition increased BSEP and ATP8B1 expression [66]. Moreover, increased bile acid production *in vivo* triggered SREBP2 and miR-33a expression to promote cholesterol synthesis and reduce CYP7A1, respectively [66].

Statins are known to increase SREBP2 and miR-33-5p expression [68], therefore, individuals taking statins will likely have consistently low levels of miR-33-5p target genes, including those involved in bile acid export, which may lead to increased hepatic cholesterol accumulation, a known side effect of statins. Indeed, mice dosed with simvastatin showed significantly lowered expression of BSEP and ATP8B1 [67]. It is possible that this could have clinical implications in which a combination of statin therapy and miR-33-5p inhibition would alleviate the hepatotoxic side effects of the drug.

Lastly, recent evidence suggests that miR-223 may also modulate CYP7A1. For example, miR-223^−/−^ mice have a nearly four-fold increase in CYP7A1 mRNA [33]. Along with miR-33-5p, miR-223 is involved in the regulation of numerous genes that maintain cholesterol homeostasis. This highlights the complexity of miRNA-mediated regulation and the importance of fully understanding the systemic consequences of miRNA inhibition or overexpression. A complete list of miRNAs involved in regulation of cholesterol transport and homeostasis is summarized in Table 1.

**Table 1:** Regulatory Actions of miRNAs and their Clinical Implications.

miRNA	Protein target(s)	Regulatory Action	Clinical Implications	References
miR-1	LXRα*	Directly suppresses LXR *in vitro*	May promote an increase in cellular cholesterol	[38]
miR-9	ACAT1*	Directly suppresses ACAT1 and esterification of cholesterol in macrophages	Overexpression may promote macrophage cholesterol efflux and reduce foam cell formation	[47]
miR-10b	ABCA1* ABCG1*	Directly represses ABCA1 and ABCG1 expression and decreases macrophage cholesterol efflux	Can be suppressed by dietary anthocyanins, leading to increased macrophage cholesterol efflux and lesion regression	[63]
miR-19b	ABCA1*	Directly suppresses ABCA1 and decreases cholesterol efflux to ApoA1; increases atherosclerotic lesion area and severity	Inhibition may increase macrophage ABCA1, promoting cholesterol efflux and lesion regression	[53]
miR-26	ABCA1* ARL7	Activated by LXR to suppress both proteins, decreasing macrophage cholesterol efflux	Inhibition may increase macrophage ABCA1, promoting cholesterol efflux and lesion regression	[58]
miR-27a/b	ABCA1* ABCG1 ACAT1* CD36 LPL*	Directly suppresses ABCA1, indirectly suppresses ABCG1, and reduces cholesterol efflux. Reduces macrophage cholesterol uptake by suppressing LPL (directly) and CD36 (indirectly), and ACAT1 (directly)	Inhibition of miR-27 would promote macrophage cholesterol efflux, but may also increase macrophage cholesterol uptake and retention	[46]
	ANGPTL3*	Directly suppresses ANGPTL3, leading to decreased EL activity	Overexpression may provide a means of increasing plasma HDL	[28]
miR-30c	MTP* ApoB	Directly suppresses MTP expression, leading to reduced ApoB secretion and formation of TRL	No hepatic steatosis, reduced TRL, fewer and smaller aortic lesions	[27]
miR-33a/b	ABCA1*	Directly suppresses ABCA1 mRNA and protein expression, decreases cholesterol efflux to ApoA1, and decreases HDL	miR-33 inhibition may increase cholesterol efflux and plasma HDL and promote regression of atherosclerotic lesions	[55,56,62,69,70]
	ABCG5/G8 ATP8B1* BSEP*	Directly suppresses ATP8B1 and BSEP, indirectly suppresses ABCG5/G8	Inhibition of miR-33 may counteract the known hepatotoxic side effects of statins	[66,67]
	CYP7A1* CYP8B1* NTCP*	Suppresses CYP7A1, CYP8B1, and NTCP, and decreases bile acid pool size	miR-33 inhibition may increase conversion of cholesterol to bile acids, reducing plasma TC	[66]
miR-33-3p	ABCA1 ABCG1	Indirectly suppresses expression of ABCA1 and ABCG1, decreasing cholesterol efflux	miR-33* inhibition may promote cholesterol efflux and lesion regression	[6]
miR-96	SRB1*	Directly suppresses hepatic SRB1 expression but increases HDL uptake	Overexpression increases hepatic HDL uptake	[32]
miR-122	ACC1* ACC2* FASN* SCD1*	Directly upregulates expression of key lipogenic genes, therefore elevating plasma cholesterol	Inhibition of miR-122 may reduce lipogenesis and subsequent release of TRL	[26]
miR-128	ABCA1* ABCG1* HMGCoA-R HMGCoA-S1 LDLR SREBP	Directly suppresses ABCA1, and ABCG1, and increases SREBP2 and its downstream genes to increase cellular cholesterol	Inhibition may decrease cellular cholesterol and promote cholesterol efflux	[49]
miR-144-5p/miR-144-3p	ABCA1*	Directly suppresses ABCA1 expression, plasma HDL, and increases atherosclerotic burden	Inhibition of miR-144/miR-144* may increase ABCA1 and promote cholesterol efflux from lesional macrophages	[5,54]
miR-145	ABCA1*	Directly suppresses ABCA1 and decreases cholesterol efflux to ApoA1	Inhibition may increase hepatic ABCA1 and promote HDL biogenesis	[50]
miR-185	SREBP2*	Directly suppresses expression of SREBP2 and its downstream genes to increase cellular cholesterol	miR-185 overexpression may decrease cholesterol biosynthesis	[35]
	SRB1*	Negatively regulates hepatic SRB1 expression and HDL uptake	Overexpression reduces HDL uptake, which may promote development of dysfunctional HDL	[32]
miR-197	HMGCoA-R HMGCoA-S1 IDI1	Indirectly increases cholesterol synthesis by binding to transcription factor FOXJ2	Suppression may decrease cholesterol synthesis	[42]
miR-206	LXRα*	Directly suppresses LXR *in vitro*	May promote an increase in cellular cholesterol	[38]
miR-223	ABCA1 CYP7A1 HMGCoA-S1* SC4MOL*	Indirectly increases ABCA1 and CYP7A1, and decreases cholesterol synthesis	miR-223 agonists may promote cholesterol efflux and HDL formation and reduce cellular cholesterol	[33]
	SRB1*	Negatively regulates hepatic SRB1 expression and HDL uptake	Overexpression reduces HDL uptake, which may promote development of dysfunctional HDL	[32,33]
miR-302a	ABCA1*	Directly represses ABCA1 expression and reduces cholesterol efflux to ApoA1	Inhibition increases hepatic and macrophage ABCA1, promoting cholesterol efflux and lesion regression	[51]
miR-378	ABCG1*	Directly decreases expression and attenuates cholesterol efflux to HDL	Antagonism of miR-378 increases ABCG1 and promotes macrophage cholesterol efflux to HDL	[64]
miR-613	ABCA1*	Directly suppresses ABCA1, attenuating macrophage cholesterol efflux to ApoA1	Suppression increases macrophage cholesterol efflux and may promote lesion regression	[61]
	LXRα*	Suppresses LXRα as part of an autofeedback loop to maintain cellular cholesterol homeostasis	May be a target for the modulation of cellular cholesterol homeostasis and LXR-target genes	[36,61]
miR-758	ABCA1*	Directly suppresses ABCA1 and attenuates cholesterol efflux to ApoA1	Suppression increases macrophage and hepatic ABCA1 and may promote cholesterol efflux	[48]

Abbreviations: ACC1: acetyl-CoA carboxylase 1; ACC2: acetyl-coA carboxylase 2; FASN: fatty acid synthase; SCD1: stearoyl-CoA desaturase 1. * indicates direct regulation by miRNA.

## 10. Dietary Influence on miRNA Expression

To further complicate this intricate gene-transcription factor-miRNA interplay, miRNAs are also targeted by certain dietary components [71]. A recent study by Wang *et al*. found that protocatechuic acid (PCA), an anthocyanin metabolite, promotes RCT by targeting miR-10b [63]. MiR-10b suppresses ABCA1 and ABCG1; *in vitro* and *in vivo* treatment with PCA suppressed miR-10b, increased expression of ABCA1 and ABCG1, and increased macrophage cholesterol efflux [63]. This study adds yet another miRNA to the list of ABCA1 regulators and also suggests a means by which certain nutrients may exert anti-atherosclerotic effects.

Milenkovic *et al*. [72] found that *in vivo* supplementation with nine different polyphenols modulated the expression of some combination of miRNAs, including miR-10b, miR-30, miR-144, miR-197, and miR-370, all of which are regulators of cholesterol metabolism [5,27,42,63,73]. However, it is necessary to consider that this study used ApoE^−/−^ mice, and knockout of ApoE alone drastically altered the miRNA profile of the animals [72]. Therefore, the changes observed following polyphenol supplementation are not translatable to wild type animals. Regardless, the authors conclude that polyphenol-induced changes in miRNA in the ApoE^−/−^ animals resulted in a relative normalization of expression patterns to resemble that of the wild type mice, suggesting that the beneficial effects of polyphenols may be mediated through miRNAs [72].

Coenzyme Q10 (CoQ10), an antioxidant and anti-inflammatory vitamin-like compound [74], was recently shown to enhance RCT by targeting miR-378 [64]. *In vitro* treatment with CoQ10 significantly increased cholesterol efflux to HDL by increasing ABCG1 expression. This increase was mediated by miR-378, such that CoQ10 reverses miR-378-mediated inhibition of ABCG1, thus promoting cholesterol efflux. The authors go on to show that suppression of miR-378 by CoQ10 is mediated by the transcription factors c-Jun and activator protein 1 (AP-1) [64]. *In vivo* supplementation with CoQ10 led to decreased expression of c-Jun and miR-378, increased ABCG1, macrophage cholesterol efflux, and RCT, and regression of aortic lesions [64].

Altogether, these data support the ability of CoQ10 to promote RCT by targeting miR-378. This is of particular importance because biosynthesis of CoQ10 shares many of the same steps as that of cholesterol biosynthesis. Therefore, statins also decrease synthesis of CoQ10, which may lead to increased expression of miR-378 and, therefore, lower ABCG1. This, once again, demonstrates the tight network of regulation of lipid metabolism and brings to light a potential side effect of statin treatment.

## 11. Conclusions

It is clear that miRNAs are involved in the regulation of most, if not all, aspects of lipid metabolism. Though it is beyond the scope of this review, miRNAs have also been implicated in regulation of lipogenesis [9,73], fatty acid oxidation [9,69], and adipose lipid metabolism [75]. Moreover, expression of many miRNAs appears to be subject to regulation by certain dietary patterns. Indeed, high-fat diets [28,32], high-cholesterol diets [48], and hypercholesterolemia [35,48] yield altered miRNA expression profiles compared with controls.

Although human-based miRNA data is lacking, an examination of plasma from individuals who had experienced an acute myocardial infarction (AMI) compared with healthy matched controls uncovered a negative association between miR-144-3p and plasma HDL and positive associations between miR-144 = 3p and plasma glucose, creatine kinase, lactate dehydrogenase, and aspartate aminotransferase, all of which are markers for AMI [5]. This suggests a potential for utilizing miR-144-3p as a marker for AMI, though it is unclear whether elevated miR-144-3p is a risk for AMI or if miR-144-3p is elevated because the individuals have had an AMI.

Though the mechanism(s) of action and complete list of target genes of each miRNA are not fully known, it has long been established that cholesterol metabolism is under very tight control. MiRNAs have recently come to light as regulators of lipid metabolism and are, therefore, potential therapeutic targets for the many lipid-related disorders plaguing the world nowadays. Cellular and animal studies show beneficial impacts associated with the promotion or inhibition of miRNA expression. However, caution should be exercised. The fact that miRNAs regulate numerous genes and that one gene can be under the regulation of numerous miRNAs suggests a complicated network of gene regulation that is not yet completely understood. In addition, some miRNAs regulate expression of other miRNAs [73]. Therefore, there is high potential for unforeseen side effects associated with miRNA-based treatments.

Nevertheless, the results of existing studies are promising. Research into the complexities of miRNA regulation of gene expression is an emerging area and as further insight is gained, there is great potential for development of dietary or drug-related treatments for disorders of lipid metabolism that target miRNAs and may lack the many side effects associated with existing therapeutic strategies.
